# Surface Sensitive Techniques for Advanced Characterization of Luminescent Materials

**DOI:** 10.3390/ma10080906

**Published:** 2017-08-04

**Authors:** Hendrik C. Swart

**Affiliations:** Department of Physics, University of the Free State, P.O. Box 339, Bloemfontein ZA93002, South Africa; swarthc@ufs.ac.za; Tel.: +27-514012926

**Keywords:** AES, XPS, TOF-SIMS, HRTEM, CL degradation, valence state, phosphors

## Abstract

The important role of surface sensitive characterization techniques such as Auger electron spectroscopy (AES), X-ray photo electron spectroscopy (XPS), time of flight scanning ion mass spectrometry (TOF-SIMS) and High resolution transmission electron microscopy (HRTEM) for the characterization of different phosphor materials is discussed in this short review by giving selective examples from previous obtained results. AES is used to monitor surface reactions during electron bombardment and also to determine the elemental composition of the surfaces of the materials, while XPS and TOF-SIMS are used for determining the surface chemical composition and valence state of the dopants. The role of XPS to determine the presence of defects in the phosphor matrix is also stated with the different examples. The role of HRTEM in combination with Energy dispersive spectroscopy (EDS) for nanoparticle characterization is also pointed out.

## 1. Introduction

With the development of flat panel displays (FPDs) and light emitting diodes (LEDs), phosphor materials became an integral part of our life style. Luminescent compounds and materials also have numerous other uses such as temperature sensors, radiation dosimeters, optical probes, storage phosphor imaging, medical imaging, microscopy techniques, homeland security, detecting tools of biological structures and can possibly be used as efficient improvement of solar cells [1,2,3]. The emission properties, whether of a fast decay rate fluorescent material or a slow decay rate phosphorescent material, depend on the chemical composition of the host and the physical structure thereof as well as the dopants and defect concentration of the luminescent material [1]. Material synthesis conditions, binder characteristics, absorbing dyes usage, etc. also play important roles in the luminescent properties. The host, valence state and crystal field are the most important properties of phosphors to be reckoned with in the design of new phosphor materials [4,5,6,7,8]. The crystal field due to the local host environment in combination with the dopant ion with the correct valence/oxidation state can be used to obtain emissions from the Ultra violet (UV) to the Infra-red (IR) wavelength ranges.

It remains important to be able to experimentally verify the oxidation state of the rare earth (RE) ions [9]. As an example, Ce^3+^ ions are good activators as well as useful sensitizers [10]. On the other hand, the possibility of Ce ions being found in the tetravalent Ce^4+^ oxidation state, which is non-luminescent, creates some difficulties in using these ions as luminescent centers in phosphors. Similar to Ce, the lanthanide Eu can also occur in two different valence states (Eu^2+^/Eu^3+^), and, although both of these are luminescent, their emission properties are very different. Eu^2+^ has emission from an allowed d-f transition, which is host dependent, whereas Eu^3+^ has emission from forbidden f-f transitions at wavelengths that do not vary with the host material.

Several analytical methods have been reported for low-level monitoring of RE ions in various sample matrices [11]. These methods include inductively coupled plasma mass spectroscopy (ICP-MS) [12], inductively coupled plasma atomic emission spectroscopy (ICP-AES) [13], isotope dilution mass spectrometry [14], resonance light scattering (RLS) [15], voltammetry [16], capillary electrophoresis [17], X-ray fluorescence (XRF) [18], fluorimetry [19], ion-microprobe [20] and other methodologies [21].

The stability of these phosphors under electron or photon irradiation is important for the FPD and LED market. The effects of bombarding with electrons vary from the development of charge, to quenching of the radiative decay of the activator excited states, to the dissociation of the gaseous or surface absorbed species causing the growth of oxide or carbon films on the surface (i.e., development of a surface “dead layer”) [22], creating defects in the surface and interface layers. Abrams and Holloway [23] discussed the development of the “dead layers”, defined to be a surface layer with no or weak luminescence. The molecular dissociation, surface chemical reactions and desorption of neutral and/or ionic atoms and/or molecules by electron or photon primary beams have been known since the 1950s to be important in surface and vacuum physics [24]. For example, electron-stimulated-desorption (ESD) was first observed because of its effects on the accuracy of vacuum pressure measurements using a hot filament ionization of a high vacuum gauge. ESD and many similar phenomena resulting from electron and photon interactions with solid surfaces are described in the book by Redhead et al. [25]. Duvenhage et al. [26] showed a 60% decrease in luminescence intensity upon UV light exposure of a mer-tris-8-hydroxy-quinolinato-indium (III) complex used in organic light emitting diodes (OLEDs). This was an indication that oxygen in moisture in the air caused some of the phenoxide rings in the complex to decompose and destroy the luminescent centers in the process. Surface characterization techniques play a vital role in the complete understanding of the luminescent properties and oxidation states of phosphor materials [8]. Auger electron spectroscopy (AES), X-ray photo electron spectroscopy (XPS), time of flight scanning ion mass spectrometry (TOF-SIMS) and High resolution transmission electron microscope (HRTEM) are used to characterize different phosphor materials. The important role of these techniques is illustrated in this paper by using example studies from previous published work.

## 2. Experimental Setup

Phosphor materials are normally prepared with different synthesized methods such as chemical bath deposition (CBD) [27], sol-gel [5,7], combustion [4], sol-gel combustion [28], hydro thermal [29], solid-state reaction [30,31], etc. Surface characterization and morphology of these phosphors are important and are carried out with AES, XPS, TOF-SIMS and HRTEM. The optical characterization is carried out with photoluminescence (PL), cathodoluminescence (CL), UV-Vis, Fourier transformed infra-red (FTIR) and Raman spectroscopy. The stability and degradation of these phosphors are important properties that are studied on a regular basis, especially for applications in display technologies.

### 2.1. CL and AES

**Figure 1:**
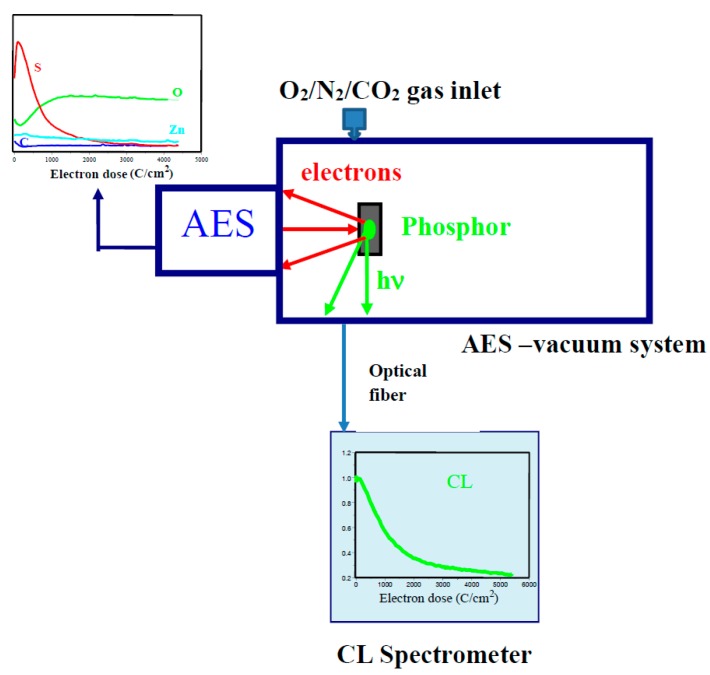
The CL/AES setup for electron degradation measurements.

Figure 1 shows the experimental setup for an in-house CL intensity degradation system. The same electron beam that is used to excite the Auger electrons of the phosphor surface is simultaneously been used to excite the phosphor material. Basically, the changes in intensity of the different CL peaks and Auger peak to peak heights (APPH) are monitored and compared with each other. The system consists of an AES vacuum system coupled with a CL spectrometer (Ocean Optics, Inc., Dunedin, FL, USA). The PHI (model 549) Auger spectrometer (Physical Electronics, INC. (PHI), Chanhassen, MN, USA) and Ocean Optics (S2000) spectrometer (Ocean Optics, Inc., Dunedin, FL, USA) are simultaneously used to collect the Auger and CL data, respectively. The light output is collected via a fiber optic connected to the CL spectrometer. The primary AES electron beam current can be adjusted and is typically between 6 and 12 µA. The beam size is varied from 100 to 300 µm depending on the focus of the beam and the beam voltage and beam current. Beam densities vary between 2.5 and 88 mA/cm^2^. The Auger and CL data are collected in a vacuum chamber with an initial base pressure in the low 10^−9^ Torr range, where after the chamber maybe backfilled with O_2_, N_2_ or CO_2_ up to pressures of 10^−6^ Torr during the degradation study. Throughout the experiment, the Auger and CL data are recorded using the same adjustable primary electron beam of 1 to 5 keV. The decrease of the CL intensities and the surface chemical changes during prolonged electron bombardment of the phosphors is monitored continuously for periods up to 24 h at the desired different gas pressures.

### 2.2. XPS

The surface chemical changes are then determined with the use of XPS. The XPS data are collected before and after degradation to evaluate the chemical composition and electronic states of the different elements. The data are collected using the PHI 5000 Versa probe-Scanning ESCA microprobe (Physical Electronics, INC. (PHI), Chanhassen, MN, USA). Low energy Ar^+^ ion gun and low energy neutralizer electron gun are used to minimize charging on the surface. Monochromatic Al Kα radiation (*hν* = 1486.6 eV) is used as the excitation source. A 25 W, 15 kV electron beam is used to excite the X-ray beam of 100 µm diameter, that is used to analyze the different binding energy peaks (pass energy 11 eV, analyzer resolution ≤0.5 eV). Multipak version 8.2 software [32] is used to analyze the chemical elements and their electronic states using Gaussian-Lorentz fits. The valence state and site positions of the dopants are also confirmed with XPS.

### 2.3. TOF-SIMS

Furthermore, TOF-SIMS measurements were performed on a PHI TRIFT V nanoTOF (Physical Electronics, INC. (PHI), Chanhassen, MN, USA) or ION TOF-SIMS (Ion-tof GmbH, Münster, Germany) by using a pulsed Au^+^ or Bi^+^ primary ion beam, to acquire chemical images of the phosphor in both the positive and the negative secondary ion polarities. The analytical field-of-view is typically 200 µm × 200 µm with a 256 pixel × 256 pixel digital raster, and the primary ion dose is maintained well within the static limit for each analysis. Charge compensation is achieved with a dual-beam (≤15 eV e^−^ and ≤10 eV Ar^+^) charge neutralizer. A raw data stream file is collected to allow full post-acquisition evaluation (i.e., retrospective analysis) of the data.

### 2.4. HRTEM

HRTEM is performed by using a JEM-2100 electron microscope (JEOL, Tokyo, Japan) at a beam voltage of 200 keV. The EDS (Energy Dispersive X-ray Spectrometer, Oxford Instruments plc, Oxfordshire, UK) mapping and chemical composition analysis is performed with an Oxford XMax 80 EDS detector (Oxford Instruments plc, Oxfordshire, UK). The ImageJ software (Laboratory for Optical and Computational Instrumentation, Wisconsin-Madison, WI, USA) is used to determine the size of the nanoparticles (NPs) from the obtained SEM/TEM micrographs. PL is done with a Cary Eclipse (Agilent, Santa Clara, CA, USA) equipped with a 150 W Xenon lamp and a 325 nm He-Cd laser. Raman spectra are obtained by using a NXR FT-Raman module microscopy (Thermo Fisher Scientific Inc, Madison, WI, USA) system with a 2.5 W Nd: YVO_4_ laser excitation of a 1064 nm wavelength and a high-performance liquid nitrogen-cooled germanium detector.

## 3. Results and Discussion

### 3.1. Electron Degradation—AES and XPS

#### 3.1.1. ZnS

**Figure 2:**
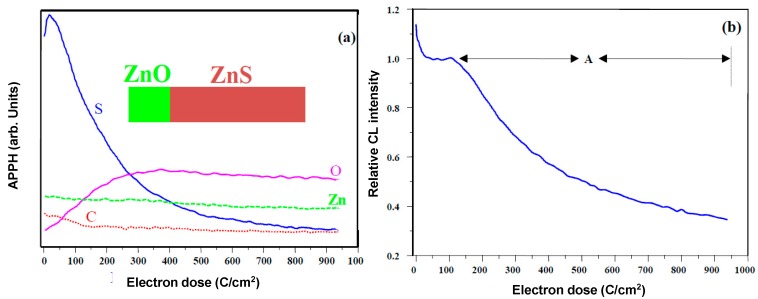
The (**a**) APPHs; and (**b**) CL intensity of ZnO during prolonged electron bombardment as function of electron dose [11].

**Figure 3:**
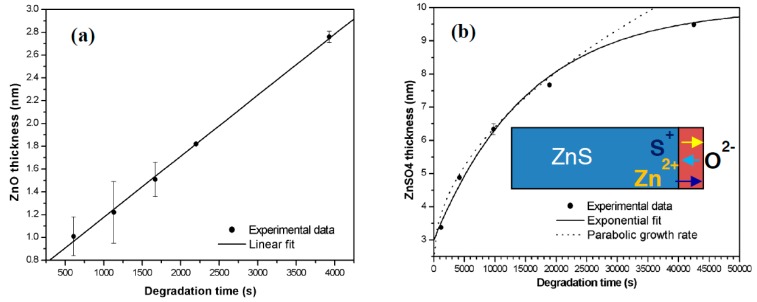
(**a**) ZnSO_4_; and (**b**) ZnO layer growth in dry and a wet oxygen atmosphere as function of degradation time with an illustration of the diffusion of the charge reactants during the ZnSO_4_ formation as an inset [35].

**Figure 4:**
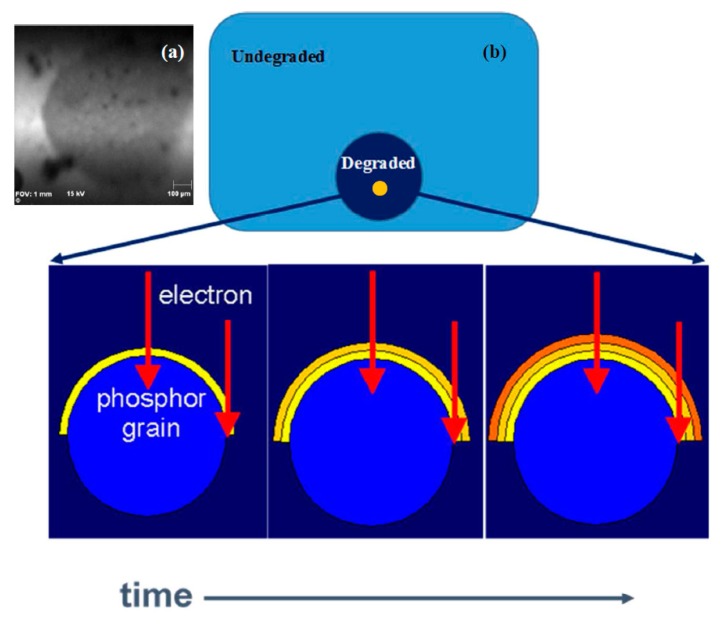
(**a**) A schematic of the degraded spot with an enlargement of one of the particles showing the growth of the degraded thin film with prolonged electron bombardment time; and (**b**) secondary X-ray Imaging (SXI) of a 24 H degraded spot of Y_2_SiO_5_:Ce.

**Figure 5:**
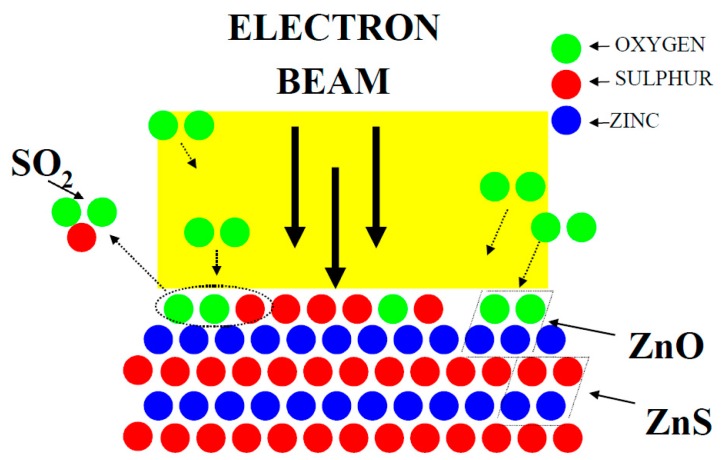
Schematic illustration of the ESSCR mechanism.

A typical electron degradation study ends up with: (a) an APPH spectrum as a function of time; and (b) a CL intensity as function of time, as demonstrated for a ZnS phosphor exposed to an electron beam as indicated in Figure 2 [33]. Figure 2a shows the APPHs of the different elements present on the surface during the degradation study, S (152 eV), C (272 eV), O (511 eV) and Zn (994 eV), against electron dose during 2 keV, 64 mA cm^−2^ electron bombardment at an oxygen pressure of 1 × 10^−6^ Torr. Oosthuizen et al. [33] found that the C APPH immediately decreased exponentially when the surface was exposed to the electron beam. The C on the phosphor surface was present from adventitious atmospheric contamination. Simultaneous to the time when the C decreased, the CL intensity stayed more or less constant (Figure 2b), which indicated that the C on the surface acted as a protected layer for the CL degradation. The S APPH at first increased due to the removal of the C from the surface and then started to decrease exponentially while the O increased after most of the C was removed from the surface. With the increase in the O and decrease in S APPHs the CL also decreased, which showed a direct correlation between the surface reactions and the CL intensity changes. It was found by Oosthuizen et al. [33] that ZnO has formed on the surface of the ZnS under prolonged electron bombardment. They also measured the presence of SO_2_ gas during electron bombardment. Itoh et al. [34], using XPS, reported that ZnSO_4_ was formed on the surface of the ZnS phosphor during electron irradiation in a H_2_O ambient. Both ZnO (−94.43 kcal/mol O_2_) and ZnSO_4_ (72.09 kcal/mol O_2_) have a negative heat of formation [33]. The degraded layer grew in thickness with prolonged electron irradiation time. Chen et al. [35] found a linear growth rate (Figure 3a) for the ZnO layer in a wet oxygen atmosphere and a ZnSO_4_ formation that decayed exponentially with time and it was postulated that this was due to the diffusion of the charge reactants through the ZnSO_4_ film to the reaction interfaces, as shown in Figure 3b. During the course of electron irradiation the bombarded areas of the phosphor became darkened in color and degraded in CL efficiency, as pointed out by the Secondary X-ray Imaging in Figure 4a. XPS was used to determine the chemical species in the degraded spot as well as on the undegraded area. A schematic of the degraded spot and growth of the non-luminescent layer with electron bombardment time is shown in Figure 4b. Holloway and Swart [36,37] came up with a well-known ESSCR (electron stimulated surface chemical reaction) mechanism that was applied to the degradation of several phosphors. According to this model, reactive gas molecules adsorb on the surface of the ZnS phosphor and are dissociated to reactive atomic species by the electron beam. This results in the formation of a non-luminescent ZnO/ZnSO_4_ layer on the surface and volatile SO_2_ as illustrated in Figure 5.

#### 3.1.2. Sr_5_(PO_4_)_3_F:Eu

**Figure 6:**
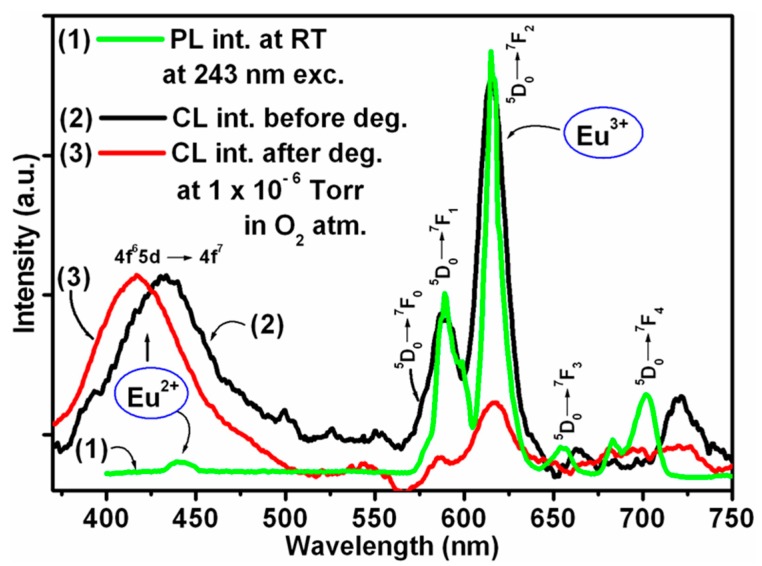
The comparative PL and CL output of the Sr_5_(PO_4_)_3_F:Eu as function of Coulomb dose at 1 × 10^−6^ Torr O_2_ atmosphere under electron beam excitation with the primary beam voltage and beam current of 2 keV and 10 μA, respectively [5].

As mentioned, other phosphors showed that the ESSCR mechanism could also be used to explain the degradation thereof. Figure 6 illustrates the comparative PL emissions of undegraded and degraded CL emission spectra of Sr_5_(PO_4_)_3_F:Eu at an oxygen pressure of 1 × 10^−6^ Torr [5]. This is a good example where the CL emission spectrum consists of both Eu^2+^ as well as Eu^3+^ emission. The Eu^2+^ wavelength position is matrix sensitive, while the Eu^3+^ emission wavelength position is not affected by a change in the matrix. The Eu^2+^ CL emission appeared as a broad band with the maxima centred at 430 nm due to the 4f^6^5d→4f^7^ transition and is more prominent compared to the PL emission. The prominent Eu^2+^ CL emission was obtained due to the blue emitting 4f^6^5d level that can be populated using high energy excitation such as cathode rays [38]. The 4f^6^5d→4f^7^ transitions of Eu^2+^ require assistance from low frequency phonons of the host matrix. In addition, the energy position of the 4f^6^5d must be blue shifted to allow population of the ^5^D_0_ state by a direct non-radiative relaxation process. The Eu^3+^ emission at 593, 616 nm due to ^5^D_0_→^7^F_1_, ^7^F_2_ transition and 578 nm due to ^5^D_0_→^7^F_0_ appeared [39] as a mirror image of the PL spectrum. After degradation, it appears that the blue emission of the Eu^2+^ ions was centered at 420 nm and remained prominent and shifted ≈10 nm compared to the undegraded sample. The CL intensity of the red emission due to the Eu^3+^ decreased drastically with an increasing in electron dose of 0 to 450 C/cm^2^. According to the ESSCR model, the Sr–O, Sr–F and P–O bonds are likely to be broken into free oxygen, fluoride, strontium and phosphorous when irradiated with a beam of electron. This will be followed by a chemical reaction resulting in new chemical compounds forming on the surface. In most cases, the new oxide layers are non-luminescent, and therefore will reduce the CL intensity. Simultaneous to the O_2_ desorption during CL degradation, it is most likely that P (metallic), SrO [40] and P_2_O_5_ [41] mixed layers were formed on the surface according to the ESSCR mechanism. The Eu^3+^ emission mostly arises from the site occupancy of the Sr_2_ metal sites which are present on the outer surface of the crystal. This caused the decrease in the Eu^3+^ CL intensity during prolonged electron exposure. The ^5^D_0_-^7^F_0_ (578 nm) and ^5^D_0_-^7^F_2_ (616 nm) Eu^3+^ emission behavior depends on the site symmetry behavior. The slight shift of the 653 and 705 nm Eu^3+^ CL emissions arise due to crystal ligand field splitting and asymmetrical environment of the crystal [39]. Simultaneously, the Eu^2+^ emission arises from the Eu ion which is present near to the defect centers, which remains protected. Thus, Eu^2+^ CL intensity remains intact during prolonged electron beam exposure. The shift in the peak position, however, indicates a change in the crystal field due to a change in the host lattice.

#### 3.1.3. Y_2_SiO_5_:Ce^3+^

**Figure 7:**
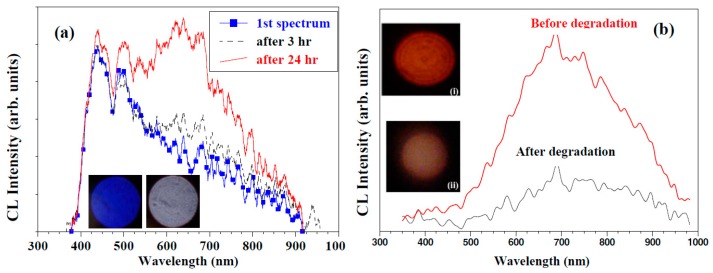
CL intensity against the wavelength for the light emitted from the powders: (**a**) before and after 3 h and 24 h electron bombardment of Y_2_SiO_5_:Ce [42]; and (**b**) SiO_2_:PbS nanoparticles before and after degradation [47] with a 2 keV electron beam at a 1 × 10^−7^ Torr O_2_ pressure. The digital images were taken before and after degradation.

**Figure 8:**
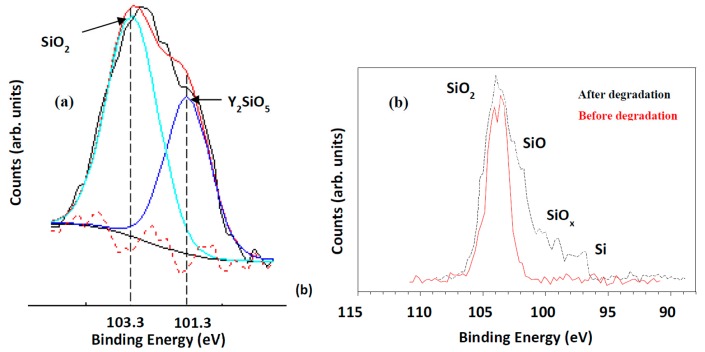
(**a**) XPS fitted results for the Si 2p in the Y_2_SiO_5_:Ce after degradation [42] 24 h degradation in an oxygen pressure of 1 × 10^−6^ Torr with the electron current density at 26.3 mA·cm^−2^; and (**b**) the XPS of SiO_2_:PbS nanoparticles before and after degradation [47] with a 2 keV electron beam at a 1 × 10^−7^ Torr O_2_ pressure.

During the degradation studies of Y_2_SiO_5_:Ce^3+^, an increase in the CL intensity at a wavelength of 650 nm was measured (Figure 7a) [42]. The XPS (Figure 8a) and CL (Figure 7a) indicated that the electron stimulated reaction led to the formation of a luminescent silicon dioxide (SiO_2_) layer on the surface of the Y_2_SiO_5_:Ce phosphor powder. The first spectrum (Figure 7a) is characteristic of the doublet character of the blue light emission from Ce^3+^, due to the 4f ground state splitting [43,44]. Figure 8a shows the fitted results from an XPS spectrum for the Si 2p peak in the Y_2_SiO_5_ state and after 24 h in the SiO_2_ state. The Si 2p peak shifted and changed shape from the yttrium silicate (Y_2_SiO_5_) chemical state with binding energy 101.3 eV to the silica (SiO_2_) chemical state 103.3 eV. The increase in CL indicates that the SiO_2_ is luminescent and thus contributing to the emission peak between 600 and 700 nm. SiO_2_ is a wide band gap phosphor material and the electron beam irradiation can break the Si–O bonds and cause intrinsic defects [45]. Skuja et al. [46] reported two peak intensities for SiO_2_ at 1.9 eV (650 nm) and 2.7 eV (459 nm) with a theory that the two peaks are related to intrinsic defects involving cleavage of the Si–O bonds. A definite contribution from the SiO_2_ 1.9 eV defect to the transition from the higher 5d levels to the 4f_7/2_ levels leads to the increase in the CL intensity and peak emission between 600 and 700 nm, thus also resulting in the change in color. The formation of the luminescent SiO_2_ layer on the surface of the Y_2_SiO_5_:Ce therefore leads to the degradation of the blue emitting Y_2_SiO_5_:Ce phosphor powders.

#### 3.1.4. SiO_2_-PbS

Figure 7b presents the broad CL spectra of SiO_2_; 0.134 mol %-PbS nanoparticles, synthesized with a sol-gel method, before and after degradation with the maximum intensity at the wavelength of about 680 nm [47]. XPS showed that during degradation in an oxygen atmosphere the SiO_2_ is reduced to SiO_x_ by the desorption of oxygen from the surface during electron bombardment and the surface was left enriched in elemental Si, as shown in Figure 8b. Dhlamini et al. [47] found that the rate of degradation of the CL intensity decreased with an increase in the oxygen pressure. The decrease in CL intensity is best explained in terms of the formation of a less efficient SiO_x_ (0 < *x* < 2) layer on the surface.

#### 3.1.5. CaO:Bi

**Figure 9:**
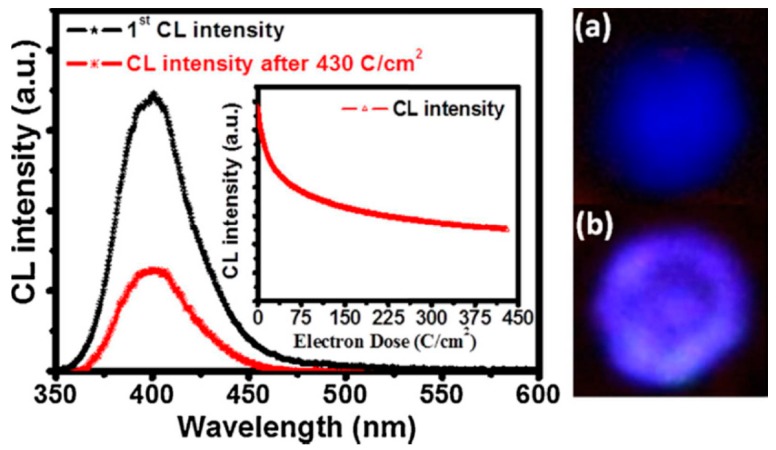
The CL emission spectra of the CaO:Bi powder before and after 430 C/cm^2^. The inset represent the CL intensity as function of prolonged electron irradiation (up to 430 C/cm^2^), with a 2 keV electron beam as working beam voltage and a 10 µA beam current. (**a**,**b**) Digital images before and after degradation, respectively, are shown [48] (Note that, although the brightness of the digital image photo (**b**) appears brighter it was a camera art effect—it was clear with the naked eye that the intensity drop severely with degradation—a change in the image of (**a**,**b**), however, is clear).

Degradation was also tested on synthesized CaO:Bi powder [48]. As indicated in Figure 9, the CL emission intensity after a 430 C/cm^2^ electron dose decreased to around 40% of the original emission intensity. The inset also represents the exponential decrease of the CL intensity as function of prolonged electron beam irradiation. The differences in color and in the homogeneous distribution of the color are clear in the digital images taken before and after degradation (Figure 9a,b, respectively). XPS [48] indicated that the degradation occurred due to ESSCRs. A Ca enriched non-luminescent surface layer formed due to the ESSCR and was responsible for the CL degradation.

#### 3.1.6. ZnO

**Figure 10:**
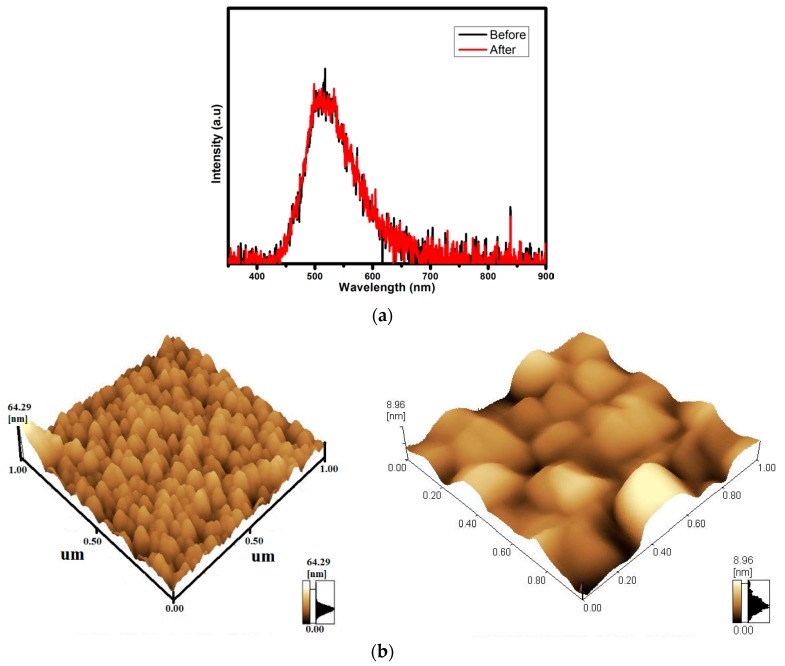
(**a**) The CL spectra before and after degradation of the ZnO film exposed to H_2_ flow for 60 min; and (**b**) AFM micrographs of the sol-gel ZnO film, measured before degradation and after degradation in the degraded area [49].

A ZnO thin film was successfully synthesized by the sol-gel method using the spin coater technique and annealed at 600 °C in air for two hours and in Ar/H_2_ (5%) flow for another 60 min [49]. CL degradation during prolonged electron irradiation on the annealed films was also determined. The CL study revealed that the intensity of the green emission (511 nm) was stable during electron bombardment for electron doses of more than 160 C/cm^2^, Figure 10a. Although the CL characteristics of the degraded sample remained the same as before degradation some surface morphology changes occurred beneath the beam during the degradation process, as can be seen in the Atomic Force Microscope (AFM) micrographs in Figure 10b [49]. It seems that a coalition of the particles occurred during prolonged electron bombardment.

#### 3.1.7. ESSCR Mechanism

In all the degradation studies, it was clear that the electron beam has to be on and gas species must be present in the degradation atmosphere for the ESSCR to occur. The degradation rate also depends on the type of gas, the current density, the temperature and the beam voltage [50]. Swart et al. [51] concluded that the surface reaction rate depends on the dissociation cross-section of the oxygen molecule and the time that an arriving oxygen molecule spends on the surface. The mean surface stay time is a function of surface temperature and decreases at higher temperatures. For the substrate atom undergoing a reaction followed by desorption, the rate of change of the surface concentration, C_s_, can be expressed as [51]:

(1) $$\frac{dC_{s}}{dt}=-kC_{s}N\emptyset_{ma}J\tau_{as}\sigma \left(\frac{\rho }{{\left(2\pi mkT\right)}^{1/2}}\right)\left(\tau_{0}exp\left(\frac{\Delta H_{des}}{kT}\right)\right)$$

where *k* is a chemical rate constant that depends on the activation energy of the chemical reaction, *Cas* is the concentration of the adsorbed atomic species that will react with the matrix, *N* is the number of reactive atomic species produced from the parent molecule and depends upon the composition of the gases, *Φ_ma_* is the dissociation cross-section of the molecules to atoms (which is a function of electron energy and current density), *J* is the electron flux density (electrons cm^−2^ s^−1^), and *τ_as_* is the lifetime of a reactive atomic species. It is assumed that the rate of production of adsorbed atomic species limits the ESSCR rate. *σ* is the sticking coefficient, *ρ* is the pressure, *m* is the molecular mass, *T* is the temperature, *k* is Boltzmann’s constant, *τ*_0_ is a combination of the molecular partition functions of the system in the equilibrium and activated states and the vibration frequency of the crystal lattice, and *H*_des_ is the desorption energy.

### 3.2. Defect Emission—XPS and HRTEM

**Figure 11:**
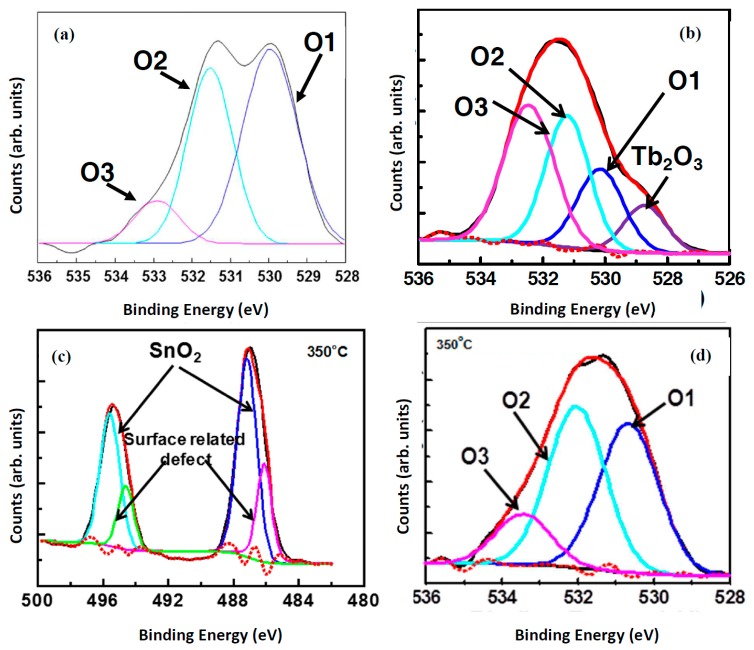
De-convolution of the O-1s peak of: (**a**) defect containing ZnO [4]; (**b**) ZnO with 6 mol % doping of Tb [45]; (**c**) Sn-3d_5/2_ and Sn-3d_3/2_ peaks of SnO_2_; and (**d**) O-1s of SnO_2_ [55].

**Figure 12:**
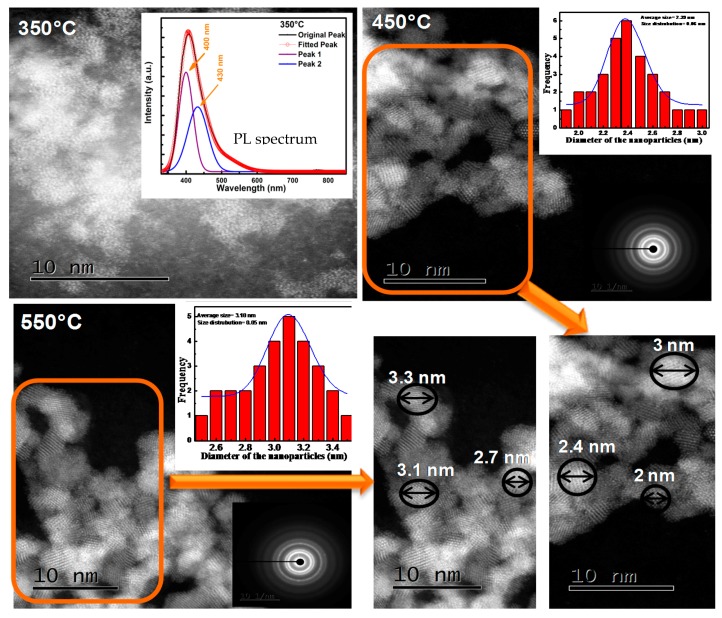
HRTEM images of SnO_2_ QDs with SAED patterns at different combustion temperatures with the 350 °C PL spectrum as an inset [55].

From the above, it is clear that the AES and XPS techniques are excellent methods to monitor the CL degradation process. XPS, however, may also be used as an indication of the presence of defects in some phosphor materials, as well as to find the oxidation state of the rare earth dopant, with the possible chemical composition thereof. Figure 11a shows the O-1s peak of a ZnO phosphor powder containing a large amount of oxygen related defects [4] and Figure 11b shows the O-1s peak of ZnO powder doped with a high concentration of Tb [52]. The defect containing ZnO was prepared by using zinc nitrate as precursors with the combustion method and the Tb (6 mol %) with the solution combustion method. Figure 11a indicates that the O-1s peak may be fitted with three peaks, namely O1 (ZnO), O2 (deficient oxygen; OH groups) and O3 (adsorbed species) centered at 530.3, 531.2 and 532.6 eV, respectively as indicated. A broad orange-red emission from 500 to 850 nm (not shown) was obtained from the ZnO prepared with the nitrate precursor which may be attributed to different kinds of defects (O*_i_*, O*_v_*, Zn*_i_* and Zn*_v_* with *i* and *v* interstitials and vacancies). The O-1s peak of the 6 mol % doping of Tb contains four peaks. The peaks are as indicated above (O1–O3) plus an extra peak at 528.7 eV which is due to the formation of Tb_2_O_3_ [53]. When Tb^3+^ ions are doped in the ZnO matrix, Tb^3+^ may occupy the sites of the Zn^2+^ ions, interstitial sites as well as the V_Zn_ sites in the ZnO lattice and emits different light color due to the newly created native defects as well as from the Tb^3+^ f-f transitions [54]. Kumar et al. [55] investigated the role of surface and deep level defects on the blue emission of tin oxide quantum dots (SnO_2_ QDs) synthesized at different temperatures by the solution-combustion method by using XPS, HRTEM and PL techniques. HRTEM revealed an increase in the average dot size from 2.2 to 3.6 nm (with Selected area electron diffraction (SAED) patterns, Figure 12) with an increasing combustion temperature from 350 to 550 °C, Figure 12. A decrease in the band gap value from 3.37 to 2.76 eV was observed with the increase in dot size due to the quantum confinement effect [55]. The PL emission showed a broad blue emission band for all the combustion temperatures studied. The 350 °C PL emission is shown as an inset in Figure 12. The fitted curve shows two peaks at 400 and 430 nm. This was due to the creation of various oxygen and Sn vacancies/defects as confirmed by X-ray photoelectron spectroscopy data (Figure 11c,d). Both Sn-3d and O-1s XPS peaks indicated the formation of defects in the SnO_2_ as indicated in Figure 11c,d) [55]. The O-1s also contains the O_2_ defect peak and the broadening of the Sn-3d peaks at the low energy side also gives an indication of the presence of the defects in the material.

### 3.3. Oxidation/Valence State—XPS

**Figure 13:**
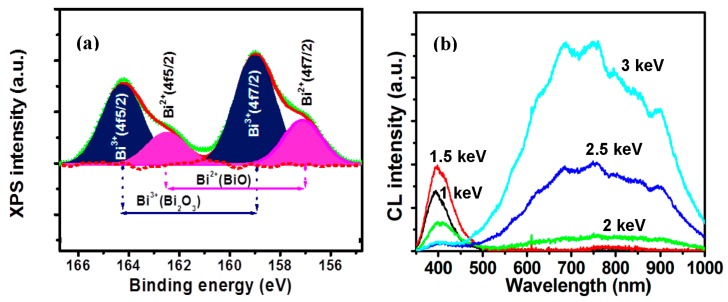
(**a**) Bi 4f high resolution deconvoluted XPS spectrum of a 1200 °C post-annealed CaO:Bi phosphor powder; and (**b**) CL spectra obtained at different beam voltages (1–3 keV) [48].

**Figure 14:**
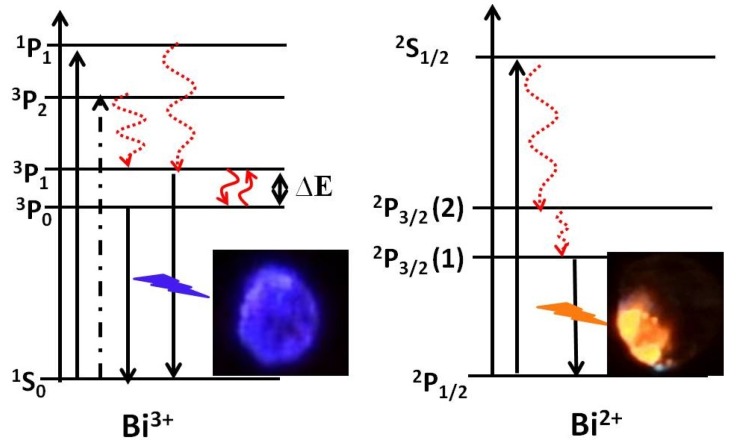
The simplified energy level diagrams of Bi^3+^ and Bi^2+^ species and the photographs of their respective luminescence [48].

A good example of using XPS for determining the oxidation/valence states is CaO:Bi. The CaO:Bi powder was synthesized by the sol-gel combustion method. The powder was then annealed in air at 1200 °C for 2 h. In order to assess the presence of the Bi in the surface layer for the sample, XPS measurements were carried out on the sample’s surface (Figure 13a) [48]. The Bi 4f XPS spectra exhibit two peaks with two shoulders which suggest that the Bi was in doublet oxidation states. The two main peaks appear with their centers at 164.3 eV and 158.9 eV, which were deconvoluted into two peaks. These peaks correspond to the Bi ^4^f_5/2_ and Bi ^4^f_7/2_ binding energies of Bi^3+^ in Bi_2_O_3_ [56]. The shoulders centered at 162.7 eV and 157.1 eV were similarly deconvoluted into two peaks. These peaks were ascribed to the Bi ^4^f_5/2_ and Bi ^4^f_7/2_ binding energies of Bi^2+^ in BiO [56]. This is evidence of the presence of the Bi with two different valence states, namely, Bi^3+^ and Bi^2+^. Figure 13b shows the CL spectra measured at different beam voltages that ranged from 1 to 3 keV. The emission changed mainly in the blue region to the orange red region with an increase in beam voltage. The blue and orange emissions were reported by many authors as a characteristic emission from Bi^3+^ and Bi^2+^ respectively [57,58]. A simplified energy level diagram, with the respected obtained color images, from Reference [48], is given in Figure 14. These diagrams explain the difference in emission between the Bi^3+^ and Bi^2+^ responsible for the different light colors obtained.

### 3.4. Oxidation/Valence State—TOF-SIMS

**Figure 15:**
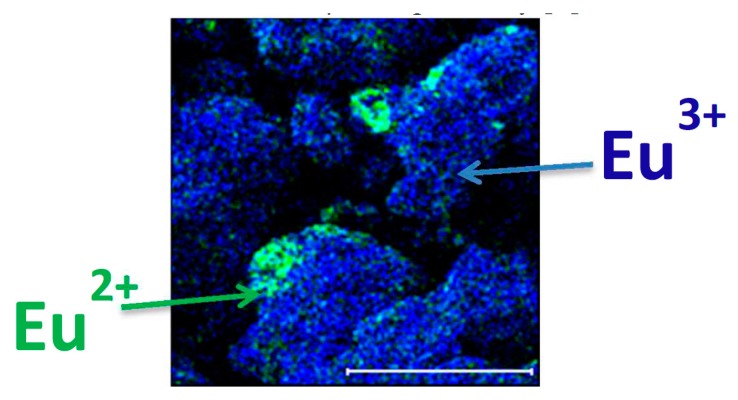
TOF-SIMS chemical imaging indicating the differences in oxidation state and distribution of the Eu^3+^ dopant and the Eu^2+^ contaminant using a false color overlay of the Eu(II)F^+^ (172 *m*/*z*; green) ion and the Eu(III)F^2+^ (191 *m*/*z*; blue) ion [59].

Normally, it is very difficult to distinguish between two oxidation states with time-of-flight secondary ion mass spectrometry (TOF-SIMS, Ion-tof GmbH, Münster, Germany), but it was shown that the technique allows full molecular and isotopic characterization of the matrix chemistry. The two states were detected in Sr_5_(PO_4_)_3_F:Eu (Figure 15) [59], by monitoring the EuF^+^ and EuF_2_^+^ species, ostensibly the Eu^2+^ and Eu^3+^ oxidation states, respectively. Ahmed et al. [60] found that at dopant concentration levels in a host material, the prospect of differentiating Ce^3+^ and Ce^4+^ ions using TOF-SIMS was poor. They found that for SiO_2_:Ce (4 mol %) samples which had been annealed in air or reduced in H_2_/Ar, that no significant differences could be detected by using TOF-SIMS, despite a difference in the Ce^3+^/Ce^4+^ ratio assessed by XPS and PL. For the best-case scenario of a Ce compound, some differences in the TOF-SIMS signals from CeF_4_ and CeF_3_ were found. Significantly, a tiny CeF^3+^ ion signal present from the CeF_4_ sample and associated with Ce^4+^ was absent from the CeF_3_ sample. This and/or differences in the relative peak sizes might be used to locate Ce^4+^ ions in fluoride materials, but using TOF-SIMS to determine the oxidation state of Ce is still challenging and more research studies are needed.

### 3.5. Surface Enhanced Raman Scattering

**Figure 16:**
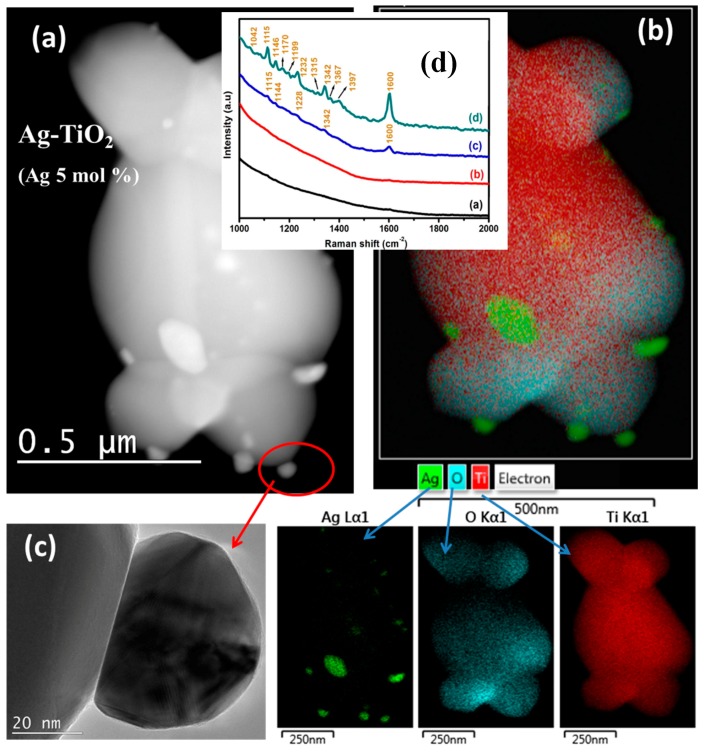
(**a**) Layered image, high-angle annular dark-field (HAADF) STEM micrograph; and (**b**) corresponding EDS mapping of Ag-TiO_2_ nanocomposites with a 5 mol % concentration (including EDS maps with HAADF micrograph). Individual EDS Maps for the elements Ag, O and Ti are shown. (**c**) HRTEM image of an Ag NP that sits on the surface of the TiO_2_ particle. Inset (**d**) shows the SERS signals of the aqueous MO solution on the surface of the: (**a**) TiO_2_; and Ag-TiO_2_ nanocomposite particles with Ag: (**b**) 1 mol %; (**c**) 3 mol %; and (**d**) 5 mol % [61].

Noble nanoparticles (NPs) combined with other optical related material such as TiO_2_ exhibit various desirable properties such as optical and antibacterial properties that make them suitable for the future nano-biotechnology and photocatalytic applications. One example is the plasmonic Ag-TiO_2_ nano-biocomposite synthesized by the sol-gel technique and their optical, surface enhanced Raman scattering (SERS). For these kinds of optical research, the HRTEM and Raman measurements are vital to explain the properties. Prakash et al. [61] found a decrease in the PL intensity with an increasing Ag NP concentration that was attributed to the decreased recombination of photo-induced electrons and holes which were trapped by the synergy in the Ag 3d energy level below the conduction band in the TiO_2_ NPs. In general, these properties such as charge separation and decreased recombination of photo-induced electrons and holes of the Ag-TiO_2_ nanocomposites are useful for improving the photocatalytic properties of the materials. The narrow emission PL bands of the Ag-TiO_2_ nanocomposites as can be seen in PL emission spectra (not shown), may additionally be useful for their application as selective optical windows [62]. Prakash et al. monitored the SERS activity of methyl orange (MO) molecules on the surface of the TiO_2_ and Ag-TiO_2_ nanocomposite particles. The inset of Figure 16 shows the SERS signals of the aqueous MO solution with TiO_2_ and Ag-TiO_2_ nanocomposite particles with an increasing Ag concentration, Figure 16 ((d)—a to d) [61]. The SERS signals become stronger at higher Ag concentrations. A full description about the stronger signal can be found in Reference [46]. The important part of the Ag-TiO_2_ as an example is the use of HRTEM in combination of EDS to indicate and found the positions of these NPs on the TiO_2_ particles (Figure 16a–c) [61]. The lighter spots on the HRTEM image are due to the Ag NPs as pointed out with EDS maps of the particle. The Ag NPSs are mostly positioned on the surface of the TiO_2_.

### 3.6. General

The examples of the results presented in this review show that the combination of AES, XPS, TOF-SIMS and HRTEM analysis can be used to obtain useful and complementary information regarding the surface and interface characteristics of phosphor materials that are complimentary to optical properties techniques. AES in combination with CL provides excellent info about the electron stimulated reactions due to electron beam radiation, while XPS and TOF-SIMS can help to determine the valence/oxidation state of the dopant and the host. XPS also may help in determining the presence of defects in the phosphor materials. Using an AES system with a focused and rastered electron beam, it is possible to obtain a secondary electron image as well as an elemental map of the same area of the sample surface in order to determine if dopants are completely dissolved into the host matrix. HRTEM in combination with EDS can indicated the positions of the doped nanoparticles into the host material. The main advantage of using the XPS-technique lies in the fact that the binding energy of a photoelectron is sensitive to the chemical surrounding of the atom’ there is a chemical shift in the binding energy. These shifts are very important since they provide a tool to identify individual chemical states of an element. Unfortunately, it is not always straightforward to interpret these chemical shifts because they depend both on initial and final state effects and then other techniques like TOF-SIMS and AES come in much more handy. It should, however, further be pointed out that ToF-SIMS is in principle is a very simple analysis technique and its high mass resolution helps to give good possibilities to identify the surface constituents of an unknown sample nut it is not always straightforward to interpret the mass spectrum due to the large amount of information obtained when a spectrum is acquired. The possibility of getting useful information from the ToF-SIMS technique increases if a known sample is measured and if used in combination with other techniques. It is then possible to draw conclusions about the structure and orientation of molecules on a surface.

## 4. Conclusions

The examples of AES, XPS, TOF-SIMS and HRTEM analysis illustrate the importance of these techniques in the development of new phosphor materials. The detection of chemical species and defects that formed on the surface and in bulk phosphor materials can help with the design of other luminescent materials. The explanation of the luminescent mechanism is much easier if the valence state of the dopant is known.
